# Alginate Microspheres Containing Temperature Sensitive Liposomes (TSL) for MR-Guided Embolization and Triggered Release of Doxorubicin

**DOI:** 10.1371/journal.pone.0141626

**Published:** 2015-11-11

**Authors:** Merel van Elk, Burcin Ozbakir, Angelique D. Barten-Rijbroek, Gert Storm, Frank Nijsen, Wim E. Hennink, Tina Vermonden, Roel Deckers

**Affiliations:** 1 Department of Pharmaceutics, Utrecht Institute for Pharmaceutical Sciences, Utrecht University, Utrecht, the Netherlands; 2 Imaging Division, University Medical Center Utrecht, Utrecht, the Netherlands; 3 Department of Radiology and Nuclear Medicine, University Medical Center Utrecht, Utrecht, the Netherlands; Brandeis University, UNITED STATES

## Abstract

**Objective:**

The objective of this study was to develop and characterize alginate microspheres suitable for embolization with on-demand triggered doxorubicin (DOX) release and whereby the microspheres as well as the drug releasing process can be visualized *in vivo* using MRI.

**Methods and Findings:**

For this purpose, barium crosslinked alginate microspheres were loaded with temperature sensitive liposomes (TSL/TSL-Ba-ms), which release their payload upon mild hyperthermia. These TSL contained DOX and [Gd(HPDO3A)(H_2_O)], a T_1_ MRI contrast agent, for real time visualization of the release. Empty alginate microspheres crosslinked with holmium ions (T_2_* MRI contrast agent, Ho-ms) were mixed with TSL-Ba-ms to allow microsphere visualization. TSL-Ba-ms and Ho-ms were prepared with a homemade spray device and sized by sieving. Encapsulation of TSL in barium crosslinked microspheres changed the triggered release properties only slightly: 95% of the loaded DOX was released from free TSL vs. 86% release for TSL-Ba-ms within 30 seconds in 50% FBS at 42°C. TSL-Ba-ms (76 ± 41 μm) and Ho-ms (64 ± 29 μm) had a comparable size, which most likely will result in a similar *in vivo* tissue distribution after an i.v. co-injection and therefore Ho-ms can be used as tracer for the TSL-Ba-ms. MR imaging of a TSL-Ba-ms and Ho-ms mixture (ratio 95:5) before and after hyperthermia allowed *in vitro* and *in vivo* visualization of microsphere deposition (T_2_*-weighted images) as well as temperature-triggered release (T_1_-weighted images). The [Gd(HPDO3A)(H_2_O)] release and clusters of microspheres containing holmium ions were visualized in a VX_2_ tumor model in a rabbit using MRI.

**Conclusions:**

In conclusion, these TSL-Ba-ms and Ho-ms are promising systems for real-time, MR-guided embolization and triggered release of drugs *in vivo*.

## Introduction

Liver cancer is the fifth most frequently diagnosed cancer in men and the second most frequent cause of cancer death, worldwide. Among primary liver cancers, hepatocellular carcinoma (HCC) represents the major histological subtype, accounting for 70 to 85% of the total liver cancer burden worldwide [1]. More than 700.000 cases of this malignant disease are diagnosed yearly with a five year survival of less than 5% [2]. Nowadays, surgical resection is the primary curative therapy for patients with HCC. Unfortunately, only 20–35% of the patients are eligible for partial resection or liver transplantation and the recurrence rate is high [3–5]. Additionally, systemic chemotherapy is mostly ineffective for these patients because only low drug concentrations are reached in the tumor, while the treatment results in severe adverse events and patient morbidity [2,6].

Transarterial chemoembolization (TACE) is a more effective treatment modality than systemic chemotherapy and principally used for inoperable HCC patients [6,7]. During the TACE procedure, a catheter is positioned in the arterial supply of a tumor via which a chemotherapeutic drug is administered followed by embolic particles [8,9]. These embolic particles block the feeding vessels of the tumor and restrict nutrient and oxygen supply to the tumor cells. In addition, the blockage of the blood vessels by the embolic particles reduces the washout of the chemotherapeutic drug, which results in higher drug concentrations in the tumor area (i.e. increased efficacy) and reduced systemic exposure (i.e. less side effects) [9,10].

Recently, drug eluting beads (DEBs), which consist of embolic particles with sizes ranging from 70–150 μm to 500–700 μm and loaded with a chemotherapeutic drug, have been developed for TACE procedure [9,11,12], delivering the embolic particles and the chemotherapeutic drugs at once [13–15]. One of the clinically used DEB formulations is based on crosslinked polyvinyl alcohol (PVA) modified with sulfonic acid groups to obtain negatively charged microspheres (DC beads^®^). A high drug concentration of positively charged cytostatics such as doxorubicin and irinotecan can be achieved in these PVA DEBs via an ion exchange mechanism [12,16,17]. Clinical trials have shown that embolization with DEBs leads to a significant reduction in peak plasma concentrations of doxorubicin [18,19] while increasing the antitumor efficacy compared to conventional TACE [20,21].

A drawback of these clinically used DEBs is, however, that they lack the ability to be visualized both during and after administration. Therefore, it is not possible to monitor the microsphere distribution in the tumor tissue, which is likely a very important factor for the treatment efficacy. Furthermore, the beads which are currently used for chemoembolization display a sustained release profile leading to low drug concentrations in the tumor over long periods of time (weeks) [17,22]. In contrast, it was recently shown by several studies that rapid intravascular release of doxorubicin from temperature sensitive liposomes leads to high intravascular drug concentrations, which subsequently enhances the drug penetration into the tumor [23–25]. This approach may lead to 20–30 times higher total drug deposition in tumor tissue compared to free drug administration and 5 times more than a Doxil-like formulation [26,27] thereby improving antitumor efficacy [26,28].

**Fig 1:**
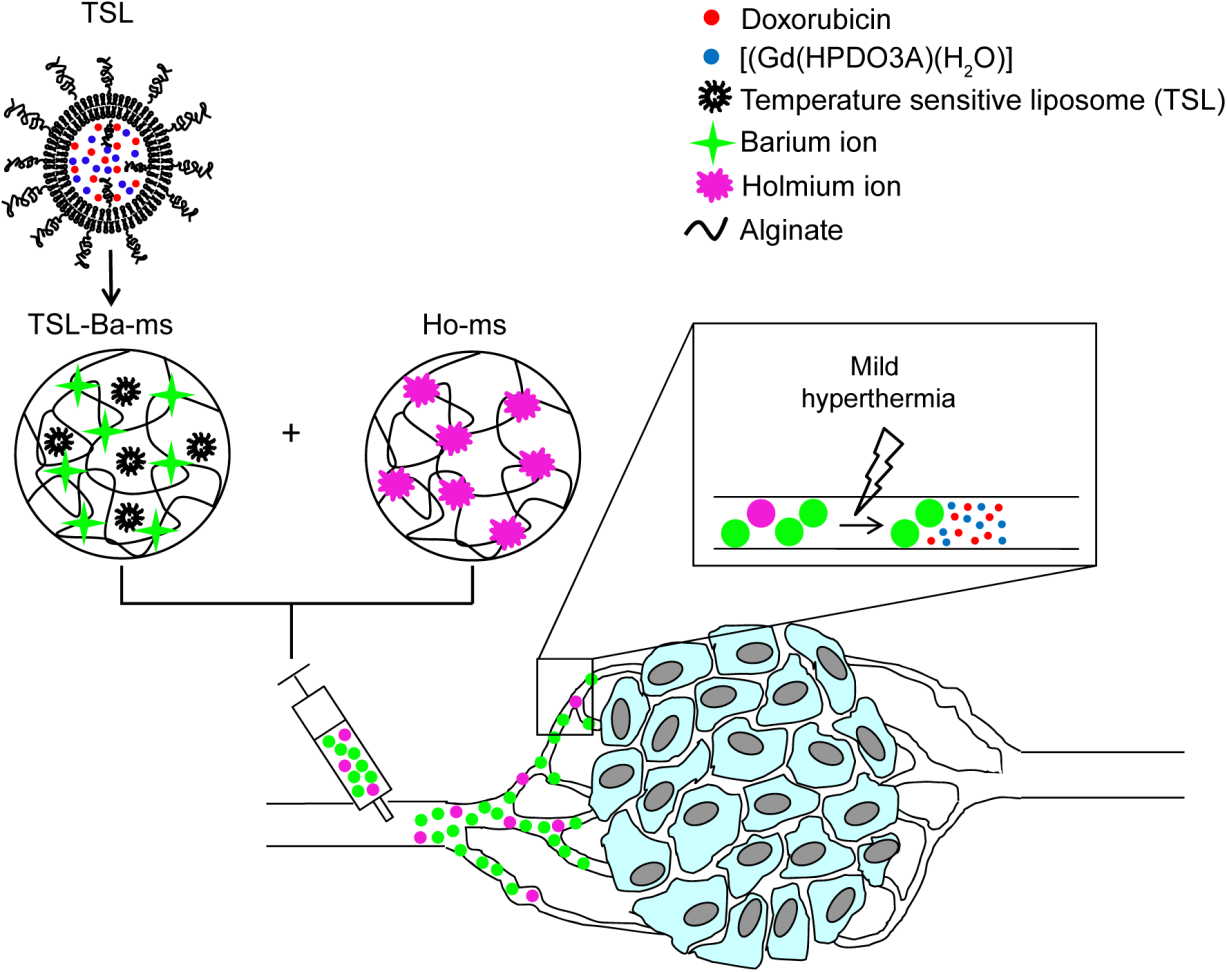
Schematic representation of temperature sensitive liposomes (TSL) loaded in alginate microspheres crosslinked with barium ions (TSL-Ba-ms). The TSL are loaded with doxorubicin (DOX) and [Gd(HPDO3A)(H_2_O)] (T_1_ MRI contrast agent). The DOX and [Gd(HPDO3A)(H_2_O)] are released from the TSL-Ba-ms during mild hyperthermia. The release of [Gd(HPDO3A)(H_2_O)] can be monitored by MRI. Empty alginate microspheres crosslinked with holmium ions (T_2_* MRI contrast agent, Ho-ms) are co-injected with TSL-Ba-ms to allow microsphere visualization by MRI.

Therefore, the objective of this study was to develop and characterize microspheres that combine embolization with on-demand temperature triggered drug release and whereby the microspheres as well as the drug releasing process can be visualized. To this end, we previously encapsulated temperature sensitive liposomes in alginate microspheres [29]. Alginate microspheres have been used for arterial embolization [30,31] and showed to be excellent embolization agents in the short term as well as the long term [28,31,32]. Furthermore, MR imaging agents were incorporated in the microspheres (i.e. holmium ions; T_2_* contrast agent) as well as in the liposomes (i.e [Gd(HPDO3A)(H_2_O)]; T_1_ contrast agent) allowing the visualization of both the microspheres and the triggered drug release. In this article we take this concept to a next level by encapsulating a cytostatic drug (doxorubicin) and a contrast agent in the liposomes. Unfortunatly, the system described in our previous study [29] is not compatible with doxorubicin release since holmium ions used for crosslinking the alginate microspheres hamper doxorubicin release (see results and discussion section). Here a novel approach is presented that allows for triggered doxorubicin release as well MR image guidance of the holmium-containing microspheres. The physicochemical and imaging characteristics of the improved system, a dispersion consisting of TSL loaded in barium crosslinked alginate microspheres (TSL-Ba-ms) and empty microspheres crosslinked with holmium ions (Ho-ms), were measured in vitro (Fig 1). Moreover, the applicability of this system was evaluated *in vivo* in a VX_2_ tumor in the auricle of a New Zealand White rabbit. In this study a water bath was used for applying hyperthermia temperatures (~42°C) to the tissue, since the tumor was easy accessible. For deep lying tumors MR guided high intensity focused ultrasound (HIFU) would be the method of choice for heating the tumor [33–35].

## Materials and Methods

### Materials

The phospholipids 1,2-dipalmitoyl-*sn*-glycero-3-phosphocholine (DPPC), and 1,2-distearoyl-*sn*-glycero-3-phosphoethanolamine-*N*-[amino(polyethylene glycol)-2000] (DSPE-PEG2.000) were purchased from Lipoid GmbH, Ludwigshafen, Germany, and 1-stearoyl-2-hydroxy-*sn*-glycero-3-phosphocholine (MSPC) was obtained from Avanti Polar Lipids, Alabaster, U.S.A. Doxorubicin-HCl was purchased from Guanyu Bio-technology Co., LTD, Xi’an, China. [Gd(HPDO3A)(H_2_O)] (Prohance^®^) was acquired from Bracco Diagnostic Inc., Monroe Township, U.S.A. Barium chloride dehydrate, Triton X-100, zinc sulfate heptahydrate, fetal bovine serum and ethylenediaminetetraacetic acid disodium salt dihydrate (EDTA) were purchased from Sigma-Aldrich Chemie BV, Zwijndrecht, The Netherlands. Holmium chloride hexahydrate was obtained from Metall rare earth limited, Shenzhen, China. Sodium alginate (Manucol LKX) was a gift from FMC biopolymer, Philadelphia, U.S.A.

### Liposome preparation

Temperature sensitive liposomes (TSL) were prepared via the well-known lipid film hydration method as described previously [36,37]. DPPC, MSPC and DSPE-PEG2.000 were dissolved in chloroform (15 mL) in a molar ratio of 86:10:4. Chloroform was evaporated under reduced pressure to form a lipid film. Residual chloroform was removed overnight under a nitrogen flow. Next, the lipid film was hydrated in 20 mL 120 mM ammonium sulfate (pH 5.4) containing 375 mM [Gd(HPDO3A)(H_2_O)] at 60°C for 15 minutes (80 μmol phospholipids/mL). The liposomal dispersion was extruded through two 200 nm filters (2 times) and two 100 nm filters (8 times). The continuous phase of the liposome dispersion was substituted by 20 mM HEPES buffer pH 7.4 containing 8 g NaCl/L via a PD-10 gel filtration column. The liposomes (15 mL) were remotely loaded with doxorubicin (DOX, 15 mL, 5 mg/mL) at 37°C for 90 minutes at a pH of 7.4 [36]. Unencapsulated DOX and [Gd(HPDO3A)(H_2_O)] were removed by ultracentrifugation (125.000 g for 45 min at 4°C). The liposomes were resuspended in 20 mM HEPES buffer pH 7.4 (15 mL) at a DOX concentration of 5 mg/mL.

### Liposome characterization

Dynamic light scattering (DLS, Malvern ALV CGS-3 system) was used to determine the size of the liposomes and their polydispersity index. Intensity correlation functions were measured using a wavelength of 632.8 nm and a scattering angle of 90°. The measurements were performed in 20 mM HEPES buffer pH 7.4 at 25°C.

In order to measure the loading efficiency, liposomes were disrupted with Triton X-100 (0.1% final concentration) and the DOX concentration in the samples was determined using fluorescence measurements (excitation wavelength 485 nm, emission wavelength 600 nm FLUOstar Optima, BMG Labtech). A calibration curve of DOX from 0 to 6 μg/mL in 20 mM HEPES buffer pH 7.4 containing 0.1% Triton X-100 was used to determine the DOX concentrations in the samples.

The temperature triggered release of DOX was measured by the change in fluorescence intensity in time (3 hours) at 37 and 42°C (excitation wavelength 468 nm, emission wavelength 558 nm, Fluorolog connected to a water bath, Horiba scientific). DOX-loaded liposomes (1 μL) were added to preheated (2 mL) 20 mM HEPES buffer (pH 7.4) or 50% fetal bovine serum (FBS) at 37 or 42°C. Triton X-100 (10%, 20 μL) was added at the end of the experiment to destroy remaining liposomes and thereby release the DOX which was still encapsulated. The percentage DOX release at both temperatures was calculated using the following equation: (I_t_-I_0_)/(I_TX_-I_0_)*100 in which I_t_ is the fluorescence intensity at time t, I_0_ the intensity at the start of the experiment and I_TX_ the fluorescence intensity after addition of Triton X-100.

### Alginate microsphere preparation

**Fig 2:**
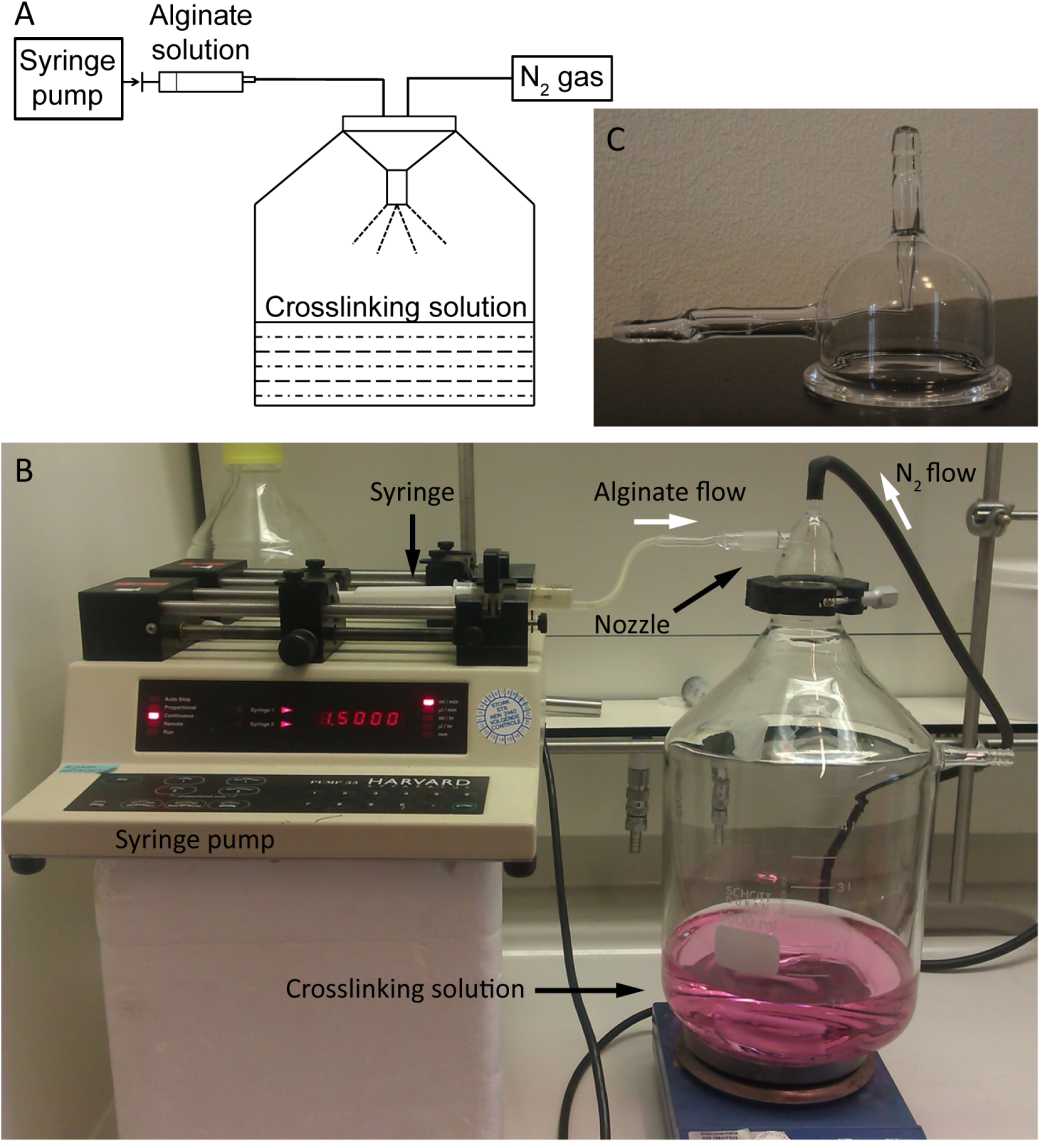
Experimental setup for the preparation of alginate microspheres. **(A) Schematic representation of the experimental setup.** An alginate solution (3% w/v in 20 mM HEPES buffer pH 7.4 containing 8 NaCl/L) is transferred into a syringe and introduced in the spraying system via a syringe pump (1.5 mL/min) which are connected via a plastic tube. Alginate droplets are formed by passing nitrogen gas (0.8 Bar) through the nozzle. The droplets are collected in a crosslinking solution (containing 100 mM barium chloride or holmium chloride). Photos of experimental setup (B) and nozzle (C).

The experimental setup for the preparation of alginate microspheres consisted of a homemade spraying device connected to a collecting vessel which is displayed in Fig 2. The spraying device consisted of a syringe pump connected to a glass nozzle via plastic tubing. Furthermore, a nitrogen flow was connected to the nozzle to induce the formation of droplets.

All types of alginate microspheres used in this study (i.e. barium crosslinked microspheres encapsulating TSL and empty microspheres crosslinked with barium ions or holmium ions) were prepared with the following protocol. An alginate solution (3% w/v in 20 mM HEPES buffer pH 7.4) was passed through the nozzle (1.5 mL/min) simultaneously with nitrogen gas (0.8 Bar) to form alginate droplets. The droplets were hardened in a crosslinking solution containing 100 mM barium chloride or 100 mM holmium chloride. The microspheres were allowed to solidify for 2 hours in the crosslinking solution and subsequently they were sized with a 300 μm sieve to remove large microspheres and aggregates. In a second sieving step, the microspheres were collected on a 50 μm sieve and washed 3 times with 20 mM HEPES buffer pH 7.4 (in total 500 mL).

Microspheres loaded with TSL were prepared by mixing 6% alginate (w/v, in 20 mM HEPES buffer pH 7.4) in a 1:1 (w/w) ratio with TSL (resulting in a final alginate solution of 3% w/v containing 40 μmol phospholipids/mL). The alginate droplets were hardened in 20 mM HEPES buffer containing 100 mM barium chloride to form TSL-Ba-ms. Empty alginate microspheres crosslinked with barium ions (Ba-ms) were prepared by spraying the alginate solution (3% w/v) in 20 mM HEPES buffer pH 7.4 containing 100 mM barium chloride. Alginate microspheres crosslinked with holmium ions (Ho-ms) were prepared by spraying the alginate solution in a crosslinking aqueous solution of 100 mM holmium chloride.

### Microsphere characterization

Morphological examination and size distribution determination of the microspheres before and after sieving were investigated with fluorescence (TSL-Ba-ms) and bright-field (Ho-ms) microscopy (BZ-9000, Keyence equipped with a GFP BP filter with an excitation wavelength of 472–30 nm and an emission wavelength of 520–35 nm and a Plan Fluor 20x lens, Nikon). The images were analyzed with calibrated BZ II Analyzer software to determine the diameter of the microspheres. The average size of the microspheres was based on the measured diameter of 500 microspheres.

In order to determine the DOX concentration in the TSL-Ba-ms, 30 mg of wet TSL-Ba-ms was disrupted in 20 mL ethylenediaminetetraacetic acid (EDTA, 5% w/v in reverse osmosis water) containing 0.1% Triton X-100. Subsequently, the DOX concentration in TSL-Ba-ms was determined by fluorescence measurements as described above.

The DOX release kinetics from TSL-Ba-ms were examined at 37 and 42°C whereby the DOX concentration in the samples was measured by UPLC. Preheated medium (20 mL, 20 mM HEPES buffer pH 7.4 or 50% FBS) at 37 or 42°C was added to TSL-Ba-ms (75 mg wet weight) and samples (1.5 mL) were taken in time. Subsequently, the samples were mixed with the same volume of cold (4°C) 20 mM HEPES buffer pH 7.4 to prevent further leakage of DOX. The samples were divided into two vials; one vial was used to determine the amount **of** DOX released and the second vial was used to determine the total amount of DOX.

Release samples (1 mL) containing FBS were centrifuged (2000 g, 5 min, 4°C) to sediment the microspheres. Acetonitrile (0.25 mL) and an aqueous solution of ZnSO_4_ (0.6 gr/mL, 35 μL) were added to the supernatant (0.75 mL) to precipitate proteins [38] and avoid clogging of the UPLC column, followed by another centrifugation step. An aqueous solution of ZnSO_4_ (0.6 gr/mL, 45 μL), acetonitrile (333 μL) and Triton X-100 (10%, 10 μL) were added to the second vial containing 1 mL sample for the determination of the total amount DOX present in the FBS samples. These samples were also centrifuged prior to UPLC measurements.

Release samples in HEPES buffer were centrifuged and 0.25 mL acetonitrile was added to 0.75 mL supernatant. EDTA (5%, 2 mL) and Triton X-100 (10%, 20 μL) were added to the second vial containing 1 mL sample for the determination of the total amount DOX. Subsequently, the samples were centrifuged and 0.25 mL acetonitrile was added to 0.75 mL supernatant.

DOX concentrations in the TSL-Ba-ms samples were determined by UPLC using a BEH C18 1.7 μm column (Waters) at 50°C and a fluorescence detector (excitation wavelength 480 nm, emission wavelength 585 nm). The eluent consisted of 1% perchloric acid and 25% acetonitrile in milli-Q water at an elution rate of 0.5 mL/min. The injection volume was 7.5 μL and the chromatographic runtime per sample was 3 minutes.

Magnetic resonance imaging (MRI) was used to visualize the Ho-ms and to monitor the temperature triggered [Gd(HPDO3A)(H_2_O)] release from the TSL-Ba-ms. Four different samples were imaged (i.e. TSL-Ba-ms:Ho-ms (95:5 and 100:0), and Ba-ms:Ho-ms (95:5 and 100:0)) before and after mild hyperthermia (15 minutes at 42°C) using the MRI sequences as described below. This allows distinguishing of the contribution of each component in this system (i.e. [Gd(HPDO3A)(H_2_O)], barium and holmium ions) on the MRI visualization of microspheres and/or [Gd(HPDO3A)(H_2_O)] release.

### Magnetic resonance imaging

All MRI experiments were performed on a clinical 1.5-Tesla MR scanner (Achieva; Philips Health care) with an 8 elements head coil (*in vitro* experiment) or a 47 mm microscopy coil (*in vivo* experiment).

The following MR sequences were used in this study: T_1_-weighted MR images were obtained using a spin echo sequence (TR = 450 ms, TE = 18 ms, FA = 90°, turbo-factor = 3, 16 slices, voxel size = 0.30x0.30x2.0 mm^3^). T_2_*-weighted MR images were obtained using a 3D gradient echo sequence (TR = 15.1 ms, TE = 9.20 ms, FA = 30°, 32 slices, voxel size = 0.30x0.30x1.0 mm^3^). Furthermore, T_1_-maps were obtained by sampling the signal recovery after inversion using a Look-Locker (LL) sequence (TR = 7.44 ms, TE = 3.5 ms, FA = 5°, turbo-factor = 5, 1 slice, voxel size = 0.80×0.80×3 mm^3^, 50 timepoints at 60 ms interval).

The images obtained from each LL measurement were automatically fitted with in-house developed Matlab software (7.12, The MathWorks Inc., Natick, MA, USA, 2000). The temporal evolution of the magnitude of the longitudinal magnetization (M) was fitted (Levenberg-Marquardt algorithm) for each pixel with the following equation:

$$M=\left|A-\left(A+B\right)\cdot e^{-t/T_{1}^{*}}\right|$$ (1)

Where T_1_* is the apparent longitudinal relaxation rate, t is the time after the inversion pulse and A and B are constants. The sample T_1_ differs from T_1_* by an offset only dependent on flip angle (α) and delay between 2 consecutive excitation pulses (Δt):

$$\frac{1}{T_{1}}=\frac{1}{T_{1}^{*}}+\text{ln}(\text{cos}\;\alpha )/\Delta t$$ (2)

Finally, T_2_*-maps were obtained by sampling the signal decay using a multi-echo gradient echo sequence (TR = 100 ms, TE1 = 8 ms, ΔTE = 8 ms, 8 echos, FA = 90°, turbo-factor = 8, 1 slice, voxel size = 0.80×0.80×3 mm^3^).

For the *in vitro* experiment (see section 2.5) the samples were placed in a sample holder containing water, which was placed in the middle of the 8 elements head coil for imaging. For the *in vivo* experiment (see section 2.8) the tumor bearing ear was placed in the middle of a 4.7 cm microcoil.

For T_1_ and T_2_* quantification one square ROI (5x5 pixels) was manually selected inside the microsphere pellet and supernatant before and after heating.

### Animal model

All experimental protocols were conducted in agreement with the Netherlands Experiments on Animals Act and the European convention guidelines, and reviewed and approved by the Animal Experiments Committee Utrecht, the Netherlands (2012.III.05.043).

Female New Zealand White rabbits (2.5–3.5 kg) were purchased from Charles River, France. All rabbits were allowed to acclimatize for at least one week before use.

VX_2_ tumor cells [39,40] were propagated in both flanks of a New Zealand White rabbit (analgesia with 4 mg/kg Carprofen^®^). The tumor was removed under analgesia and sedation (Carprofen^®^ 4 mg/kg, Dexdormitor^®^ 0.125 mg/kg and Narketan^®^ 15 mg/kg) when reaching a tumor diameter of ~ 3 cm. A VX_2_ cell suspension was generated by dissecting and fragmenting tumor tissue using a cellstrainer (Easystrainer 100 μm, Greiner). A tumor in the auricle of a New Zealand White rabbit was induced by injecting 200 μL of the cell suspension (8.5x10^8^ cells/mL PBS) subcutaneously into the auricle of the rabbit under analgesia and anesthesia (Carprofen^®^ 4 mg/kg, Dexdormitor^®^ 0.125 mg/kg and Narketan^®^ 15 mg/kg).

### In vivo imaging

When the tumor in the auricle reached a diameter of ~2 cm after 2 weeks the *in vivo* imaging study was performed. Carprofen^®^ (4 mg/kg, 0.25 mL), Dexdormitor^®^ (0.125 mg/kg, 0.75 mL) and Narketan^®^ (15 mg/kg, 0.45 mL) were administered subcutaneously for analgesia and anesthesia. The rabbit was positioned in the MRI scanner with the tumor bearing auricle in a warm water bath at 37°C. A dispersion of 152 mg TSL-Ba-ms and 8 mg Ho-ms suspended in 1 mL mM HEPES buffer pH 7.4 was injected intratumorally. Next, the water bath in which the tumor was positioned, was heated up to 46°C (such that the interior of the tumor reached at least 42°C) for 15 minutes to induce release of [Gd(HPDO3A)(H_2_O)]. T_1_ and T_2_*-weighted images and T_1_ and T_2_* maps were made (as described in section 2.6) prior to the injection of the microspheres and immediately after the intratumoral injection of the microspheres as well as after the incubation at elevated temperatures.

## Results and Discussion

### Preparation and characterization of temperature sensitive liposomes encapsulating doxorubicin (DOX) and [Gd(HPDO3A)(H_2_O)]

Temperature sensitive liposomes (TSL) containing lysolipids were prepared via the lipid film hydration method (the characteristics of the TSL are described in Table 1) [36,37]. These TSL were passively loaded with [Gd(HPDO3A)(H_2_O)] (a T_1_ MRI contrast agent) during hydration of the lipid film and subsequently remotely loaded with doxorubicin (DOX). The mean diameter of the liposomes was 134 nm with a PDI ≤ 0.1. The encapsulation efficiency of DOX was > 95% as expected for remotely loaded liposomes [36,41–43]. [Gd(HPDO3A)(H_2_O)] was passively loaded into the liposomes and has therefore an encapsulation efficiency around 10% [29].

**Table 1:** Characteristics of temperature senstitive liposomes (TSL) loaded with DOX and [Gd(HPDO3A)(H_2_O)].

	TSL
Lipid composition	DPPC:MSPC:DSPE-PEG2.000
Molar ratio lipids (feed ratio)	86:10:4
Mean diameter (PDI)	134 nm (0.09 +/- 0.02)
DOX encapsulation (%)	>99%

**Fig 3:**
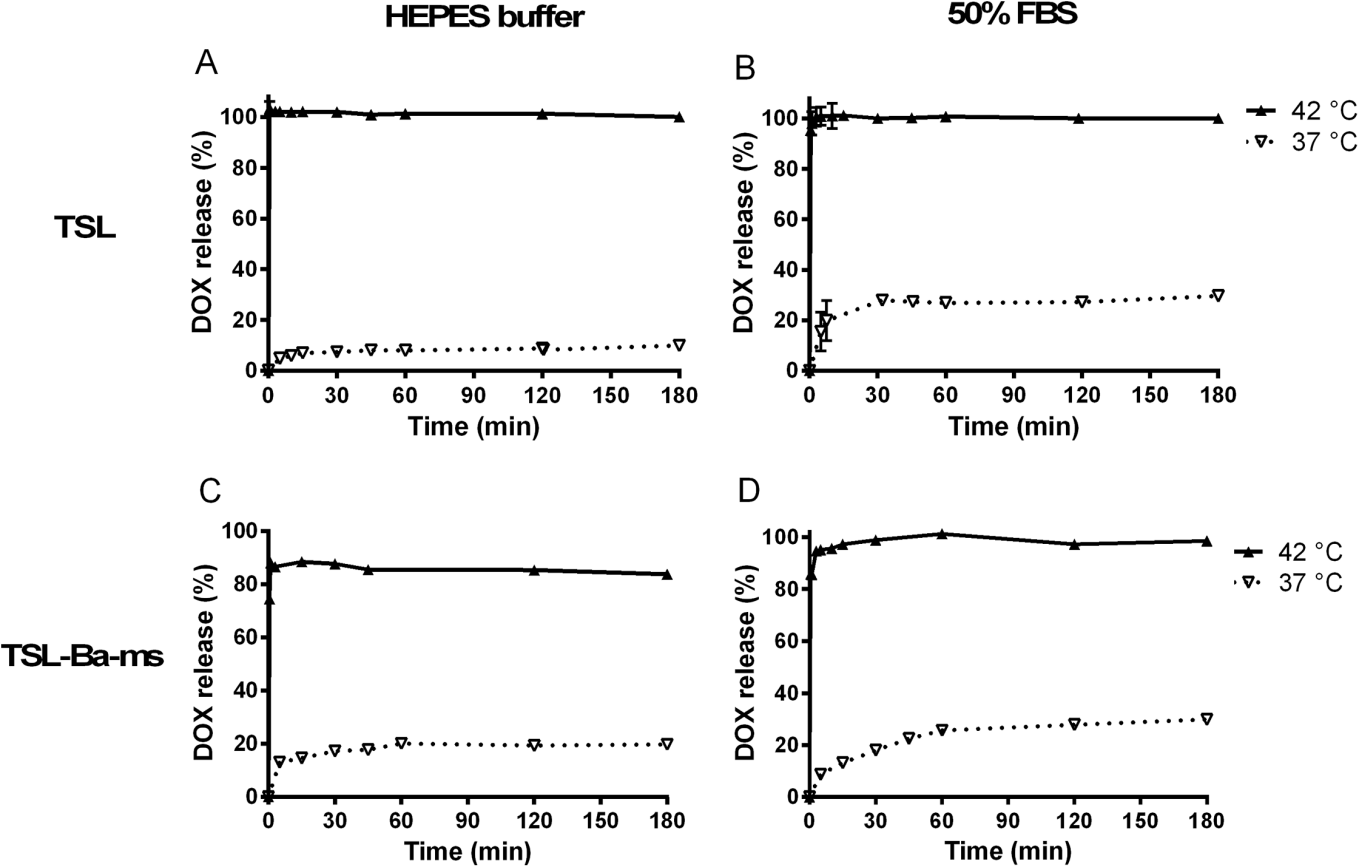
Temperature triggered release of DOX from TSL. Temperature triggered release of DOX from TSL (DPPC:MSPC:DSPE-PEG2.000 86:10:4) in 20 mM HEPES buffer pH 7.4 (A) or 50% fetal bovine serum (B) at 37 and 42°C. Temperature triggered DOX release from TSL-Ba-ms in 20 mM HEPES buffer pH 7.4 (C) and 50% FBS (D) at 37 and 42°C.

Fig 3 shows the release of DOX from TSL before encapsulation in the alginate microspheres as a function of incubation time at 37 and 42°C in 20 mM HEPES buffer pH 7.4 (Fig 3A) and in 50% fetal bovine serum (FBS) (Fig 3B), respectively. TSL released less than 10% of their content after 3 hours incubation in HEPES buffer at 37°C while at 42°C complete release took place within 30 seconds. In 50% FBS at 37°C, the TSL showed a release of ~15% within 5 minutes and reached 30% release after 3 hours. The release rate at 37°C in 50% FBS was higher than in HEPES buffer (Fig 3A and 3B) likely because proteins destabilized the lipid bilayer [44–46]. At 42°C more than 95% of the loaded DOX was released within 30 seconds in 50% FBS (Fig 3B), which is comparable with the release of DOX in HEPES buffer at the same temperature, indicating that DOX is released nearly quantitatively at 42°C independent of the presence of proteins in the release medium.

### Preparation of barium crosslinked alginate microspheres loaded with TSL (TSL-Ba-ms)

**Fig 4:**
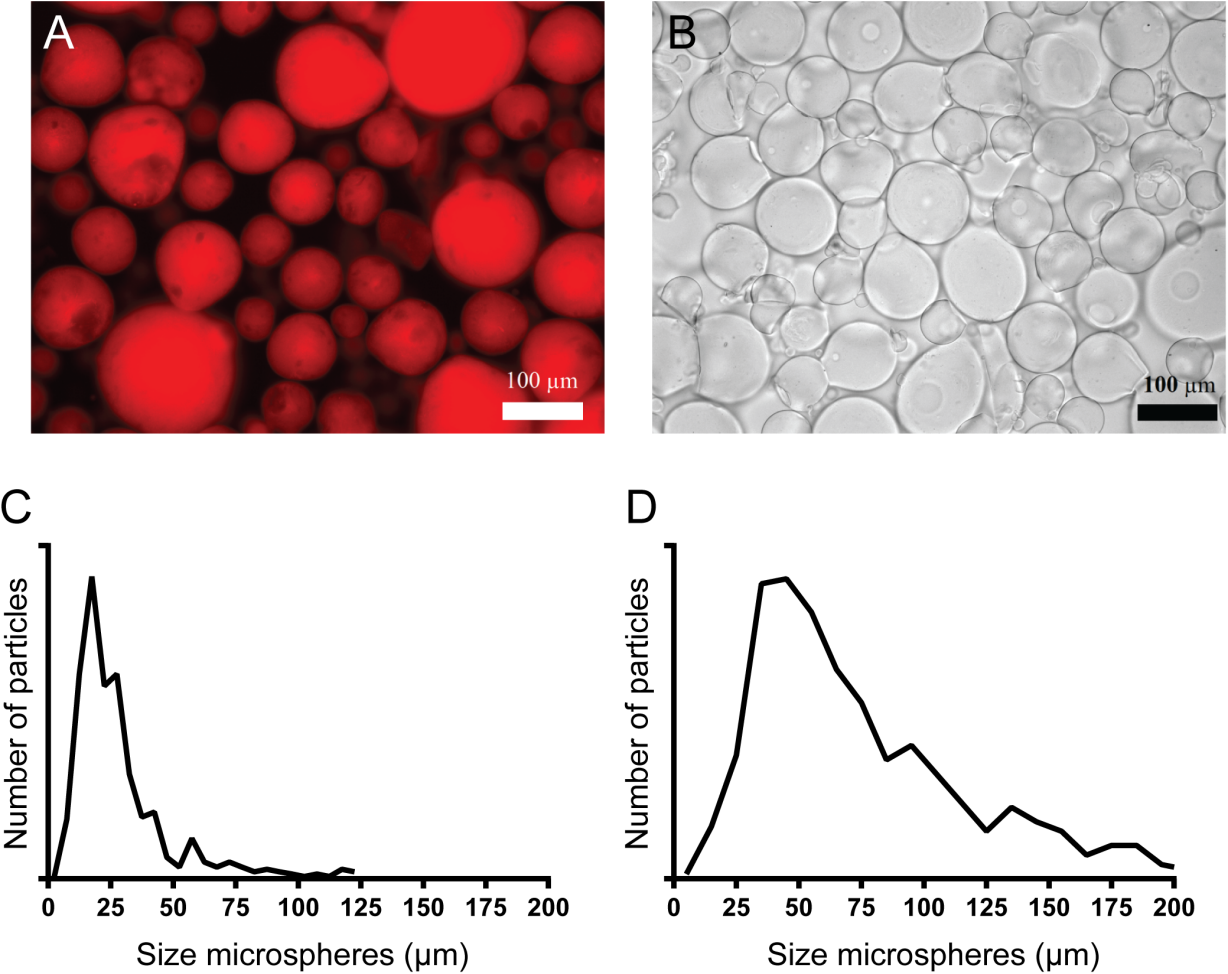
Microscopy images and size distribution of TSL-Ba-ms and Ho-ms. Fluorescence microscopy image of TSL-Ba-ms (A) and bright-field microscopy image of alginate microspheres crosslinked with holmium ions (Ho-ms) (B) after sieving. The size distribution of TSL-Ba-ms before (C) and after (D) sieving was analyzed with calibrated BZ II Analyzer software.

Alginate microspheres containing TSL were prepared with the spraying device shown in Fig 2. An alginate solution containing TSL was sprayed into 20 mM HEPES buffer pH 7.4 containing 100 mM barium chloride. The mean diameter of the unfractionated TSL-Ba-ms was 30 μm with a standard deviation of 21 μm while the mean diameter was 76 μm with a standard deviation of 41 μm after sieving (Fig 4C and 4D). This demonstrates that many microspheres had a size below 50 μm, which were subsequently successfully removed by sieving. The encapsulation efficiency of TSL into the alginate microspheres was 60% based on DOX measurements. This relatively low encapsulation efficiency is based on the instability of TSL in the alginate solution since alginate can be inserted into the lipid bilayer [47] leading to DOX leakage from the TSL before formation of the TSL-Ba-ms. The presence of DOX loaded liposomes in the alginate microspheres could also be qualitatively observed with fluorescence microscopy (Fig 4A).

### Characterization of empty alginate microspheres crosslinked with holmium ions

Empty microspheres crosslinked with solely holmium ions (Ho-ms) were prepared and sieved similar to the TSL-Ba-ms described above. This resulted in Ho-ms with a mean diameter of 64 μm and a standard deviation of 29 μm (Fig 4B), which is similar to the diameter of TSL-Ba-ms (Fig 4A). Since, the chemical composition of the Ho-ms and TSL-Ba-ms are very similar, both consist mainly of water (> 98%), the size is the dominant variable influencing their biodistribution upon intra-arterial injection [48,49]. Considering the fact that Ho-ms and TSL-Ba-ms have a comparable size, they most likely have a similar *in vivo* tissue distribution after an i.v. co-injection and therefore Ho-ms can be used as tracer for the TSL-Ba-ms. The concentration of holmium ions in the Ho-ms is relatively low, therefore the toxicity of these microspheres is expected to be low [29,30].

### Release of doxorubicin (DOX) from TSL-Ba-ms

The release of DOX from TSL-Ba-ms at 37 and 42°C in 20 mM HEPES buffer pH 7.4 (Fig 3C) and the same buffer with 50% FBS (Fig 3D) was evaluated over a time period of 3 hours. At 37°C, TSL-Ba-ms displayed a marginal release (20%) of DOX after incubation in HEPES buffer for 3 hours. At 42°C, approximately 75% of DOX was released within 30 seconds while a maximum release of 85% was achieved after 1 minute in HEPES buffer. This incomplete DOX release from TSL-Ba-ms in HEPES buffer (containing 0.8% NaCl) is most likely due to the interaction of positively charged DOX and negatively charged alginate. Addition of Triton X-100 did not lead to extra release of DOX which would be the case if some liposomes remained intact after applying mild hyperthermia. The incomplete release of DOX was also observed visually since TSL-Ba-ms remained slightly pink after incubation at 42°C.

In 50% FBS, TSL-Ba-ms showed a DOX release of 30% after 3 hours incubation at 37°C. At 42°C however, the TSL-Ba-ms released DOX nearly quantitatively within 3 minutes. The difference in DOX release found in HEPES and 50% FBS can be explained by the presence of proteins. Presumably, proteins in the release medium desorb DOX from the negatively charged alginate since it is reported in other studies that DOX can interact with plasma proteins [50,51].

Previously, we prepared microspheres containing liposomes which were crosslinked with barium as well as holmium ions in a molar ratio of 95:5 [29]. When TSL containing DOX were loaded into these microspheres, no release of DOX occurred upon mild hyperthermia (Figure A in S1 File). Likely, the released DOX interacted with the holmium ions present in the microspheres since a color shift from red to purple was observed during incubation at 42°C (Figure A in S1 File). This holmium-DOX complexation was reported previously [52,53] and also evidenced by the fact that the purple color of a solution containing DOX and holmium ions returned to red after the addition of EDTA, which captures the holmium ions (data not shown). Thus it was concluded that DOX was released from the liposomes during mild hyperthermia, however the formation of holmium ion-DOX complexes prevented the release of DOX from the microspheres.

In the current approach DOX might also interact with Ho-ms after being released from the TSL-Ba-ms. However, only 5% of the DOX was captured after 5 minutes incubation with Ho-ms (Figure B in S1 File). Furthermore, It is expected that the DOX upon release from the TSL will diffuse quickly out of the microspheres into the tumor tissue. Therefore, the influence of the Ho-ms on the efficacy of DOX will be minimal.

### Visualization of microspheres and [Gd(HPDO3A)(H_2_O)] release by MRI

As mentioned in the introduction holmium ions and [Gd(HPDO3A)(H_2_O)] are MRI contrast agents that allow visualization of the microspheres and triggered drug release, respectively. To investigate in more detail the contribution of each component in our system (i.e. barium ions, holmium ions and [Gd(HPDO3A)(H_2_O)]) on the MR signal, T_1_ and T_2_*-weighted (wt) MR images were obtained of i) TSL-Ba-ms mixed with Ho-ms (ratio 95:5), ii) TSL-Ba-ms only, iii) Ba-ms mixed with Ho-ms (ratio 95:5) and iv) Ba-ms only.

**Fig 5:**
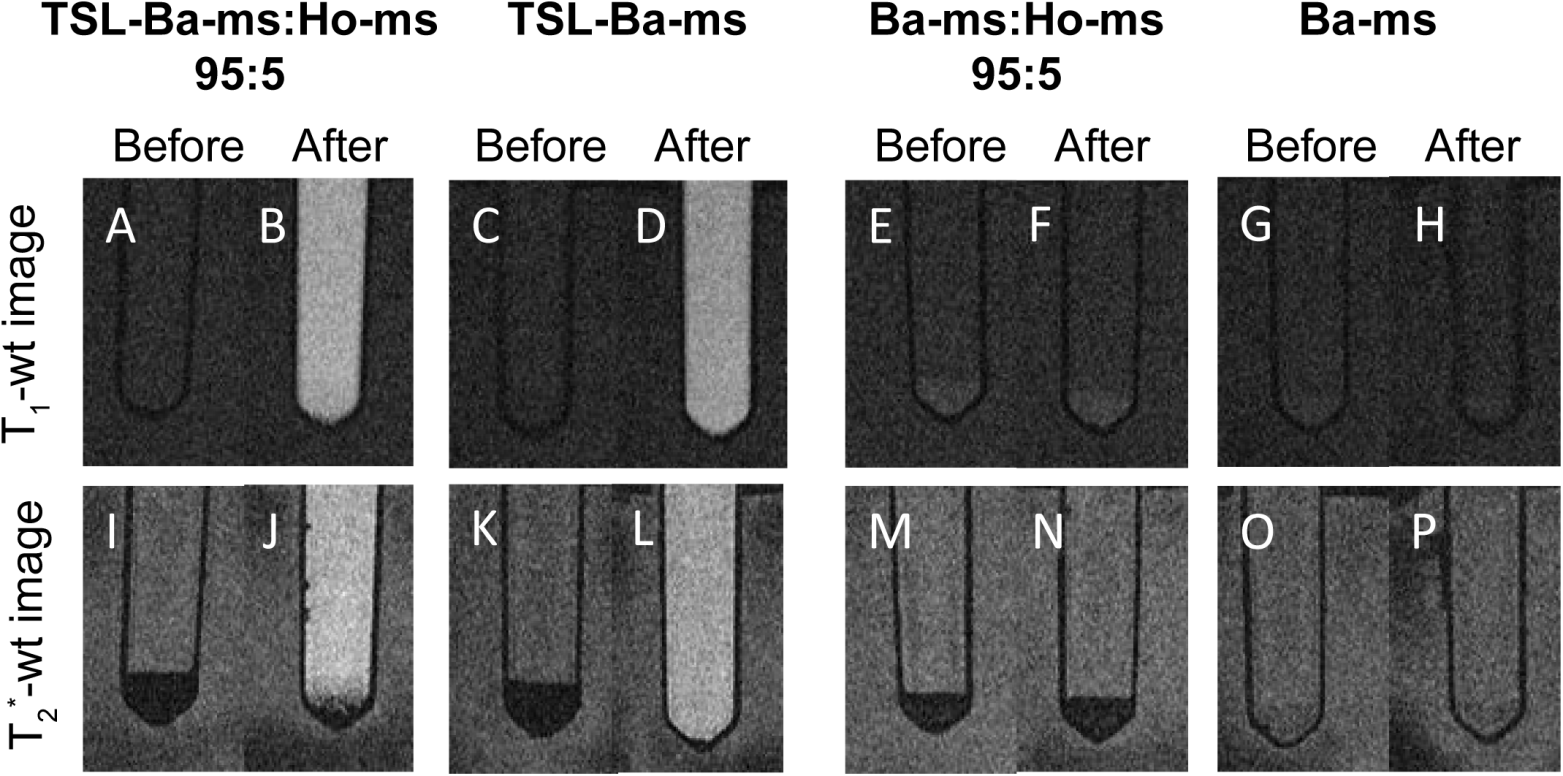
T_1_ and T_2_*-weighted MR images of TSL-Ba-ms or Ba-ms mixed with or without Ho-ms (ratio 95:5) before mild hyperthermia. T_1_ and T_2_*-weighted MR images of TSL-Ba-ms or Ba-ms mixed with or without Ho-ms (ratio 95:5) before mild hyperthermia and after incubation at 42°C for 15 minutes. Signal enhancement in the supernatant after mild hyperthermia indicates release of [Gd(HPDO3A)(H_2_O)] in the T_1_-wt image. The holmium ions appear hypointense in the T_2_*-wt image due to local distortion of the magnetic field.

Fig 5 shows T_2_*-wt and T_1_-wt MR images of these four combinations before and after incubation at mild hyperthermia (42°C for 15 minutes). The microspheres sedimented and the microspheres were therefore present in a pellet at the bottom of the tube. As expected, the holmium containing microspheres were clearly detectable as a black layer on the T_2_*-wt image before and after mild hyperthermia (Fig 5I, 5J, 5M and 5N), whereas microspheres containing barium only (Ba-ms) were not observed on T_2_*-wt images (Fig 5O and 5P). Interestingly, TSL-Ba-ms were also clearly detectable on T_2_*-wt images before hyperthermia because of the T_2_*-effect of [Gd(HPDO3A)(H_2_O)] at high concentrations (Fig 5K). However, upon temperature triggered release and subsequent dilution of [Gd(HPDO3A)(H_2_O)], the T_2_*-effect of gadolinium decreased and the TSL-Ba-ms were no longer visible on the T_2_*-wt image (Fig 5L). The temperature triggered [Gd(HPDO3A)(H_2_O)] release was best visualized on T_1_-wt images (Fig 5A–5D). These images show an increase in signal intensity in the microsphere pellet as well as in the supernatant of the TSL-Ba-ms after applying mild hyperthermia (Fig 5B and 5D). This demonstrates that MRI allows the visualization of the microspheres (containing holmium ions) as well as the release of [Gd(HPDO3A)(H_2_O)] at the same location in an *in vitro* situation.

**Fig 6:**
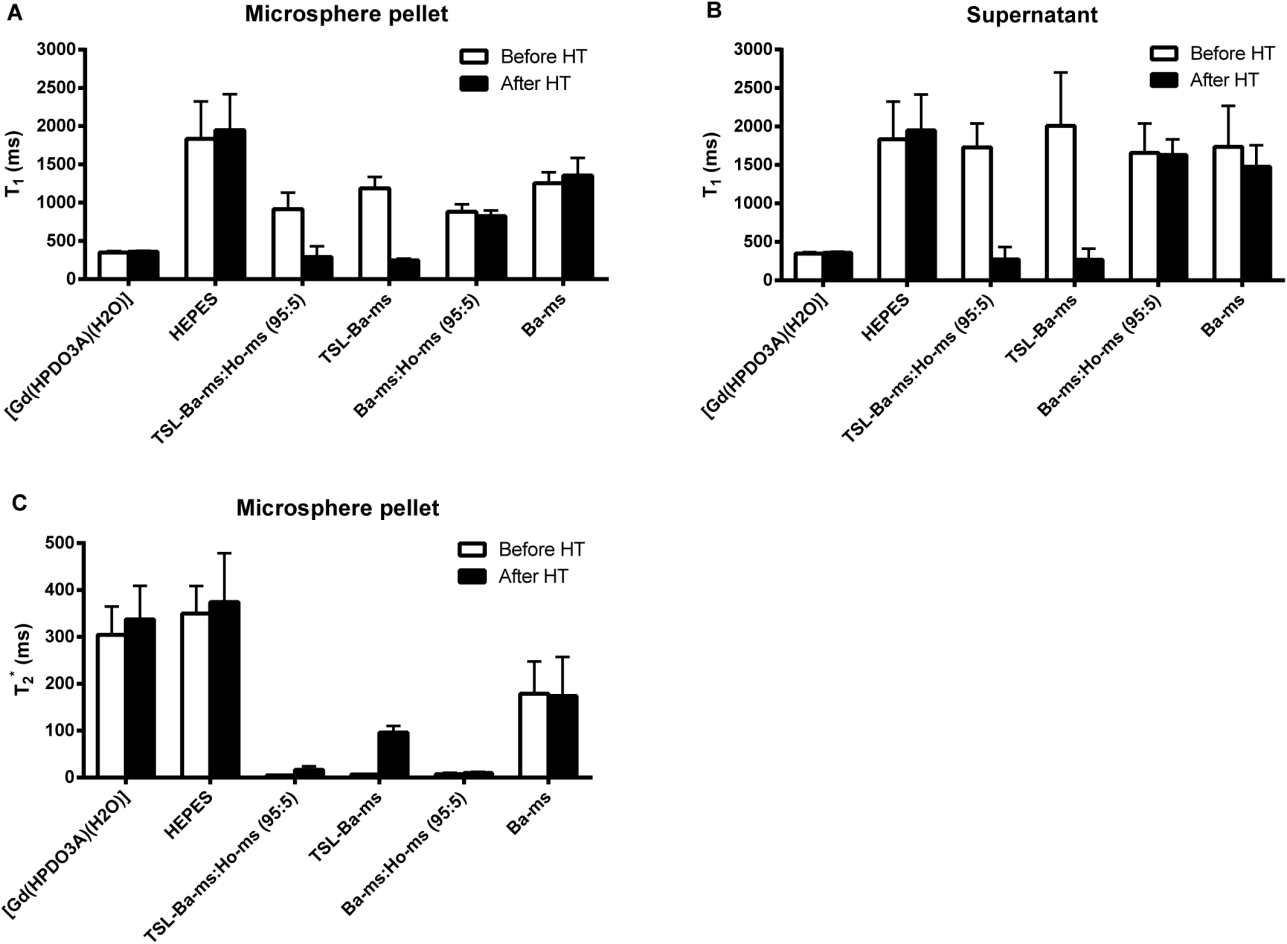
T_1_ and T_2_* in the microsphere pellet and supernatant of alginate microspheres before and after mild hyperthermia. T_1_ in the microsphere pellet (A) and supernatant (B) and T_2_* in the microsphere pellet (C) of alginate microspheres before and after mild hyperthermia (HT; 42°C for 15 minutes). A region of interested (ROI) was positioned in the microsphere pellet (if present) or the supernatant to determine the T_1_ and T_2_*-values (Mean + standard deviation).

Additionally, quantitative T_1_ and T_2_*-mapping was performed of the same samples described above (i.e TSL-Ba-ms mixed with Ho-ms (ratio 95:5), TSL-Ba-ms only, Ba-ms mixed with Ho-ms (ratio 95:5) and Ba-ms only) before and after mild hyperthermia (Fig 6). Furthermore, HEPES and a free [Gd(HPDO3A)(H_2_O)] solution, at approximately the same concentration (0.5 mM) as obtained after [Gd(HPDO3A)(H_2_O)] release from the TSL-Ba-ms, were included as control samples. Fig 6 shows the mean T_1_ and T_2_*-values in a region of interest (ROI) postioned in the microsphere pellet (if present) or the supernatant. The T_2_*-values of the supernatant are not shown because they were too long (as expected for liquids lacking macromolecules) to be adequately measured with the sequence used. The T_1_-value of the samples containing TSL-Ba-ms decreased in the microsphere pellet as well as in the supernatant after mild hyperthermia (Fig 6A and 6B). The T_1_-values of these samples after heating were the same as the T_1_ of free [Gd(HPDO3A)(H_2_O)] at the same concentration in the microsphere pellet as well as in the supernatant, indicating quantitative release of [Gd(HPDO3A)(H_2_O)] from TSL-Ba-ms after applying hyperthermia. As expected, Ba-ms did not show a change in T_1_-value before and after applying mild hyperthermia. The presence of 5% Ho-ms had only a minor effect on the T_1_-values of the microsphere pellet and the supernatant and therefore does not interfere with the detection of released [Gd(HPDO3A)(H_2_O)]. In contrast, the T_2_*-value of samples containing Ho-ms was, as expected, very short in the presence of Ho-ms before and after mild hyperthermia (Fig 6C). As explained previously, [Gd(HPDO3A)(H_2_O)] exhibits a strong T_2_*-effect at high concentrations (i.e. loaded in TSL). Therefore, the hyperthermia triggered release of [Gd(HPDO3A)(H_2_O)] caused an increase of T_2_* in the TSL-Ba-ms pellet, which confirms the necessity of Ho-ms for visualizing the microsphere distribution after applying hyperthermia, otherwise the microspheres would be no longer visible.

### In vivo visualization of the release of [Gd(HPDO3A)(H_2_O)] and the distribution of a TSL-Ba-ms and Ho-ms mixture in a VX_2_ tumor present in the auricle of a rabbit

**Fig 7:**
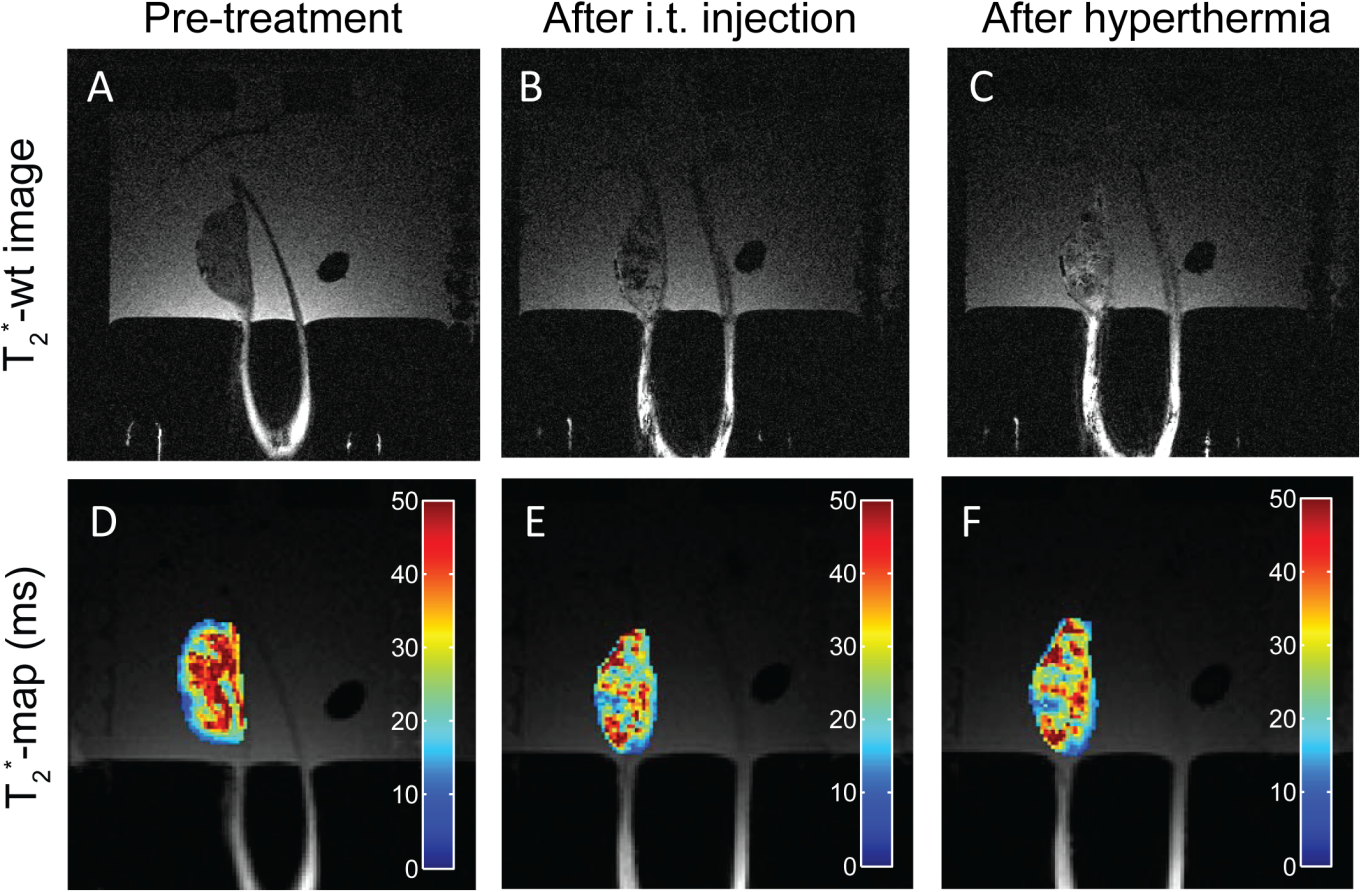
T_2_*-wt images and T_2_*-maps of intratumoral injected TSL-Ba-ms and Ho-ms in a tumor in the auricle of a New Zealand White rabbit. T_2_*-wt images (A-C) and T_2_*-maps (D-F) of a tumor in the auricle of a New Zealand White rabbit before (A, D) and after (B, E) intratumoral injection of TSL-Ba-ms and Ho-ms. Finally, the tumor was heated in the range between 42 and 46°C for 15 minutes (C, F).

The feasibility of TSL-Ba-ms and Ho-ms (in a ratio of 95:5) to allow *in vivo* monitoring of the microsphere deposition and [Gd(HPDO3A)(H_2_O)] release was evaluated in the auricle VX2 tumor of a rabbit after intratumoral injection of a dispersion containing TSL-Ba-ms as well as Ho-ms. Implanting the tumor in the auricle of a rabbit made the tumor easy accessible for hyperthermia treatment using a water bath. In addition, the hypothesis that the Ho-ms co-localize with TSL-Ba-ms and therefore can be used as a tracer for TSL-Ba-ms, was investigated. T_1_ and T_2_*-wt images and T_1_ and T_2_*-maps were made at 3 timepoints: i) before intratumoral injection of the microspheres, ii) immediately after the intratumoral co-injection of TSL-Ba-ms and Ho-ms (95:5 ratio) and iii) shortly after applying mild hyperthermia. The tumor tissue looked relatively homogeneous on the T_2_*-wt image prior to administration (Fig 7A). Intratumoral co-injection the dispersion of TSL-Ba-ms and Ho-ms (95:5) caused signal voids on the T_2_*-wt images indicating the presence of microsphere clusters in the tumor (Fig 7B). These clusters were still visible as signal voids on the T_2_*-wt images after applying mild hyperthermia for 15 minutes (Fig 7C). The T_2_*-maps showed the same response: intratumoral co-injection of the microspheres caused a shortening of the T_2_* in the tumor (Fig 7D and 7E), whereas the application of hyperthermia did not influence the T_2_* of the tumor (Fig 7E and 7F).

**Fig 8:**
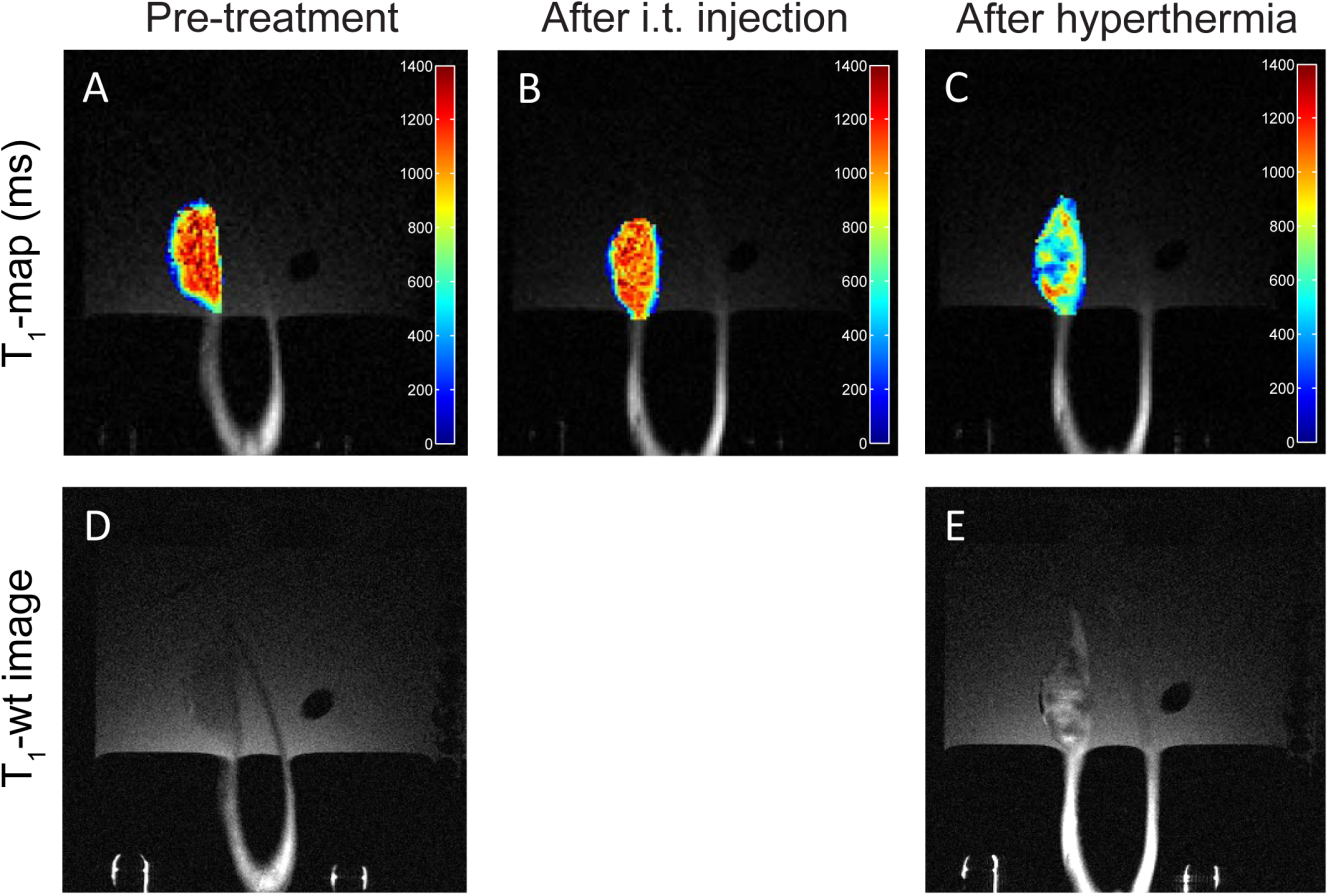
T_1_-maps and T_1_-wt images of intratumoral injected TSL-Ba-ms and Ho-ms in a tumor in the auricle of a New Zealand White rabbit. T_1_-maps (A-C) and T_1_-wt images (D-E) of a tumor in the auricle of a New Zealand White rabbit before (A,D) and after (B) intratumoral co-injection of TSL-Ba-ms and Ho-ms. Finally, the tumor was heated in the range between 42 and 46°C for 15 minutes (C,E).

No difference in tumor T_1_-values was observed after intratumoral co-injection of the microsphere mixture (Fig 8A and 8B). This indicates that [Gd(HPDO3A)(H_2_O)] was not released and thus the TSL loaded into the microspheres remained intact during the injection of the microspheres. After the intratumoral co-injection of the microspheres, the tumor was heated in the range between 42 and 46°C for 15 minutes to trigger the release of [Gd(HPDO3A)(H_2_O)] from the liposomes and subsequently from the TSL-Ba-ms. A drop in T_1_-values was observed in the tumor after applying hyperthermia, indicating release of [Gd(HPDO3A)(H_2_O)] from the TSL-Ba-ms (Fig 8C). This observation is strengthened by the fact that the intrinsic T_1_-value of tissue increase with increasing temperature [54,55].

The T_1_-wt images showed the same response: the tumor tissue looked relatively homogeneous on the T_1_-wt image prior to administration (Fig 8D), whereas the co-injection of the microspheres followed by applying hyperthermia caused a shortening in T_1_ (Fig 8E).

Furthermore, the location of Ho-ms (i.e. largest change in T_2_*-value) corresponded very closely with the location of the TSL-Ba-ms (i.e. largest change in T_1_-value) making Ho-ms a suitable tracer for TSL-Ba-ms.

## Conclusion

This paper shows that alginate microspheres encapsulating temperature sensitive liposomes containing a T_1_ MRI contrast agent ([Gd(HPDO3A)(H_2_O)]) and doxorubicin (DOX) (TSL-Ba-ms) were successfully developed. The encapsulation of TSL in Ba-ms changed the triggered release properties only slightly. Also empty holmium (T_2_* MRI contrast agent) crosslinked microspheres (Ho-ms) were prepared. The incorporation of MR imaging agents in the microspheres (i.e. holmium ions in Ho-ms) as well as in the liposomes ([Gd(HPDO3A)(H_2_O)] in TSL-Ba-ms) allowed for visualization of both the microspheres and the triggered drug release *in vitro* using MRI. The microsphere deposition and [Gd(HPDO3A)(H_2_O)] release after intratumoral co-injection of TSL-Ba-ms and Ho-ms was monitored in the VX_2_ tumor model. The deposition of Ho-ms overlapped very closely with the location of the [Gd(HPDO3A)(H_2_O)] release from TSL-Ba-ms making Ho-ms a suitable tracer for TSL-Ba-ms. Since in this study only an intratumoral injection was performed the embolization properties of our system were not characterized, though this will be addressed in a future study. Furthermore, we will investigate the antitumor efficacy of the proposed system compared to free DOX and standard TACE.

Taken together, these microspheres are attractive systems for real-time, MR-guided embolization and triggered release of drugs.

## Supporting Information

S1 File Interaction of DOX and Ho-ms after DOX-release. **Figure A** shows temperature triggered DOX release from TSL encapsulated in alginate microspheres crosslinked with barium ions and holmium ions in a 95:5 ratio. **Figure B** shows the uptake of DOX as function of incubation time with Ho-ms.
(DOCX)
