# Supplementary material for: Alginate Microspheres Containing Temperature Sensitive Liposomes (TSL) for MR-Guided Embolization and Triggered Release of Doxorubicin
Source: PLoS One. 2015 Nov 11;10(11):e0141626. doi: 10.1371/journal.pone.0141626 (PMC4641710; doi:10.1371/journal.pone.0141626)
Supplement: S1 File — Figure A shows temperature triggered DOX release from TSL encapsulated in alginate microspheres crosslinked with barium ions and holmium ions in a 95:5 ratio. Figure B shows the uptake of DOX as function of incubation time with Ho-ms. (DOCX) [file pone.0141626.s001.docx]

**Supporting Information**

**Alginate microspheres containing temperature sensitive liposomes (TSL) for MR-guided embolization and triggered release of doxorubicin**

Merel van Elk^1^, Burcin Ozbakir^1^, Angelique D. Barten-Rijbroek^2^, Gert Storm^1^, Frank Nijsen^3^, Wim E. Hennink^1^, Tina Vermonden^1^ and Roel Deckers^2*^

^1^ Department of Pharmaceutics, Utrecht Institute for Pharmaceutical Sciences, Utrecht University, Utrecht, the Netherlands

^2^ Imaging Division, University Medical Center Utrecht, Utrecht, the Netherlands

^3^ Department of Radiology and Nuclear Medicine, University Medical Center Utrecht, Utrecht, the Netherlands

**DOX release from microspheres crosslinked with barium ions and holmium ions (95:5 ratio)**


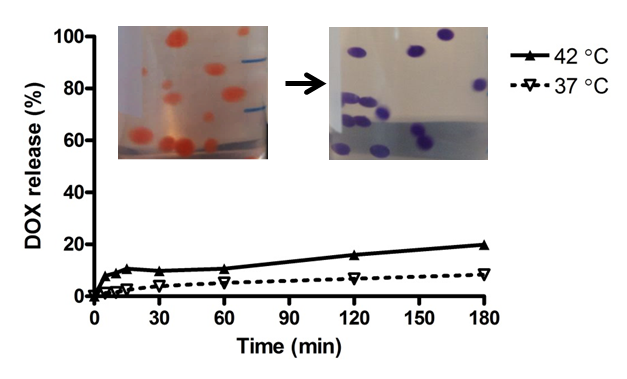


**Fig A. Temperature triggered DOX release from Ho-Ba-ms.**

Temperature triggered DOX release from TSL encapsulated in alginate microspheres crosslinked with barium ions and holmium ions in a 95:5 ratio. Release was measured in preheated 20 mM HEPES buffer pH 7.4 at 37 and 42 °C. A color shift from red to purple was observed during incubation at 42 °C.

**Interaction of DOX with Ho-ms**

DOX was dissolved at a concentration of 4 ug/mL in an aqueous buffer (20 mM HEPES buffer + 0.8% NaCL, pH 7.4) containing 8 mg Ho-ms in 40 mL. These concentrations correlate with the concentration of DOX/expected concentration of Ho-ms in our release experiment. At 0, 5, 10, 15, 30, 60, 120 and 180 minutes a sample was taken from this dispersion after sedimentation of the microspheres, the concentration of DOX was measured using fluorescence measurements (excitation wavelength 485 nm, emission wavelength 600 nm). Figure 2 shows the DOX concentration as function of incubation time with Ho-ms. After 5 minute incubation of DOX and Ho-ms, less than 5% of the DOX was taken up by the microspheres. The DOX uptake by Ho-ms gradually increases with increasing incubation towards 50% after 180 minutes. However, it is expected that the DOX upon release from the TSL will diffuse quickly out of the microspheres into the tumor tissue. Therefore, the percentage of DOX uptake by Ho-ms will be minimal.


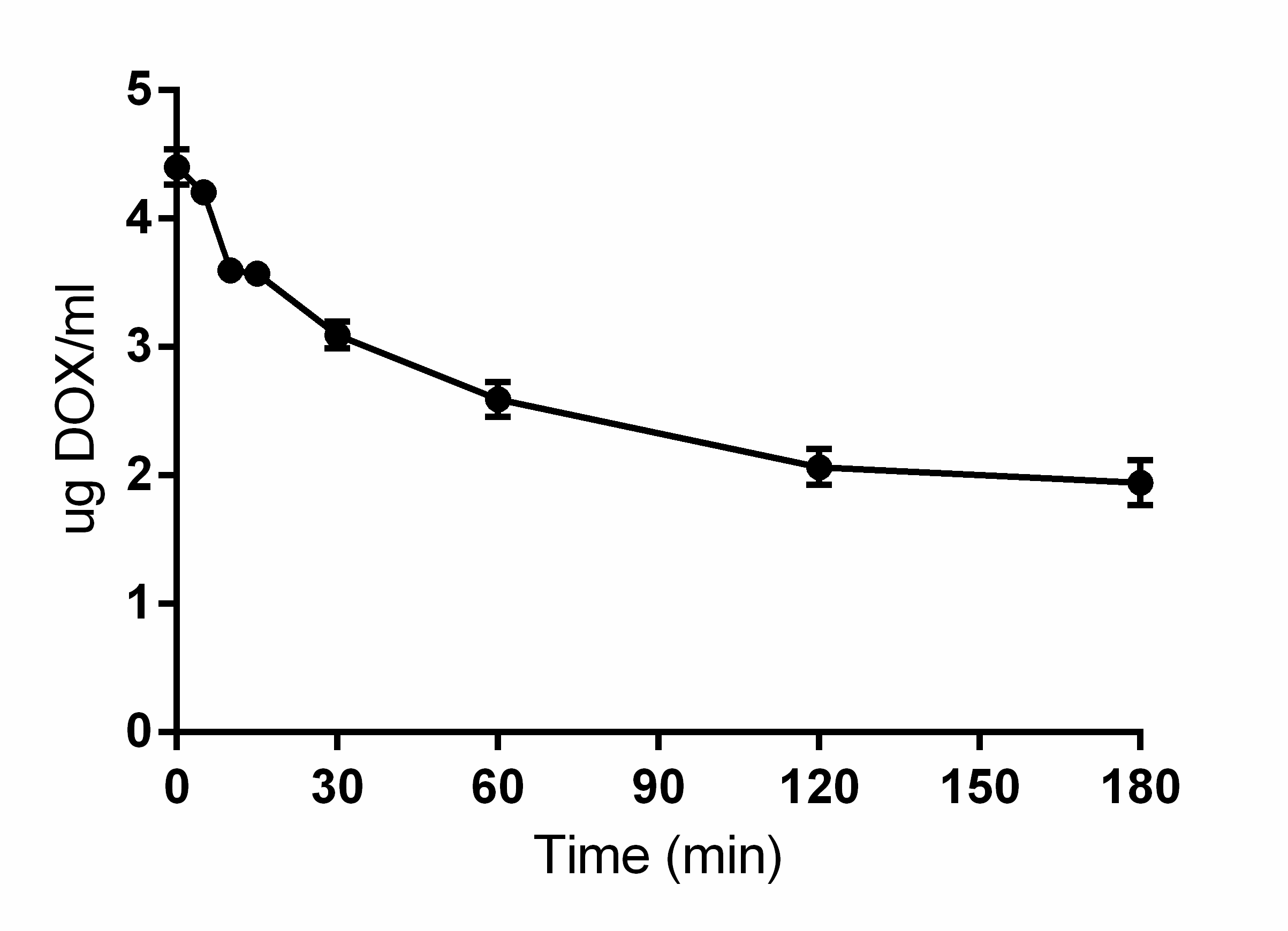


**Fig B. Free DOX concentration as function of incubation time with Ho-ms.**
